# Honey bee microRNAs respond to infection by the microsporidian parasite *Nosema ceranae*

**DOI:** 10.1038/srep17494

**Published:** 2015-12-01

**Authors:** Qiang Huang, Yanping Chen, Rui Wu Wang, Ryan S. Schwarz, Jay D. Evans

**Affiliations:** 1 State Key Laboratory of Genetic Resources and Evolution, Kunming Institute of Zoology, Chinese Academy of Science, Kunming, 650223, China; 2 USDA-ARS Bee Research Laboratory, BARC-East Building 306, Beltsville, Maryland, 20705, USA

## Abstract

In order to study the effects of *Nosema ceranae* infection on honey bee microRNA (miRNA) expression, we deep-sequenced honey bee miRNAs daily across a full 6-day parasite reproduction cycle. Seventeen miRNAs were differentially expressed in honey bees infected by *N. ceranae* that potentially target over 400 genes predicted to primarily involve ion binding, signaling, the nucleus, transmembrane transport, and DNA binding. Based on Enzyme Code analysis, nine biological pathways were identified by screening target genes against the Kyoto Encyclopedia of Genes and Genomes (KEGG) database, seven of which involved metabolism. Our results suggest that differentially expressed miRNAs regulate metabolism related genes of host honey bees in response to *N. ceranae* infection.

MicroRNAs (miRNAs) are a family of endogenous RNAs that regulate gene expression in a sequence-specific manner. miRNAs are usually 22 nucleotides long with a seed region (2–8 bp) that binds to the 3’ untranslated region (UTR) of a targeted mRNA, signaling it for degradation. miRNAs have diverse expression patterns and regulate developmental and physiological processes^1^. More recently, miRNAs have been reported as a virulence factor of fungal parasites of plants^2^.

*Nosema ceranae* is a unicellular fungal parasite that infects mid-gut epithelial cells of honey bees^3^,^4^. The intracellular reproduction cycle of *N. ceranae* lasts approximately four days and the parasite starts to reproduce daughter spores soon after entering epithelial cells^5^. The infected cell eventually bursts, releasing a large number of spores. *Nosema* infection can profoundly affect the global gene expression and physiology of honey bee hosts^6^,^7^. Genome-wide quantitative trait locus (QTL) association analysis identified a honey bee gene, *Aubergine*, that might be involved in suppressing *N. ceranae* reproduction^8^. *Aubergine* belongs to the *Argonaute* family, genes encoding key proteins involved with the RNA-induced silencing complex (RISC)^9^. RISC directs small regulatory RNAs, such as miRNAs and small interfering RNAs (siRNAs), to cleave target mRNAs^10^. In order to study the impacts of *N. ceranae* infection on honey bee miRNAs, we infected honey bees with *N. ceranae* spores and monitored miRNA expression changes at 24 hour intervals for a complete infective cycle (6 days post infection). With this time series analysis, we provide new insight into mechanisms of microsporidian pathogenesis and honey bee responses.

## Results

### Differentially expressed honey bee miRNAs during *N. ceranae* infection

Successful infection by *N. ceranae* was confirmed by screening two parasite genes (NCER_101240 and NCER_100079) with RT-qPCR (Table S1). 134 mature miRNAs were detected during the infection period (Additional file 1). Out of those, 17 were differentially expressed in infected honey bee workers compared with control workers (Table 1). Six miRNAs were differentially expressed at two time points and the rest were significant at one time point. For 134 detected miRNAs, a statistically significant association between up or down regulation with post infection day was not detected (χ^2^ = 5.733, df = 5, p > 0.05). However, the 17 differentially expressed miRNAs were not randomly distributed across time points (χ^2^ = 11.52, df = 3, p < 0.05; Table 1). Five of the 17 differentially expressed miRNAs (ame-miR-12, ame-miR-315, ame-miR-317, ame-miR-31a, ame-miR34) were used to verify the accuracy of RNA sequencing data with RT-qPCR. Expression levels of these selected miRNAs were significantly correlated between the two techniques over 6 days post infection (R = 0.674, p < 0.05) (Fig. S1).

### Functional analysis of target genes of significantly differentially expressed miRNAs

**Figure 1:**
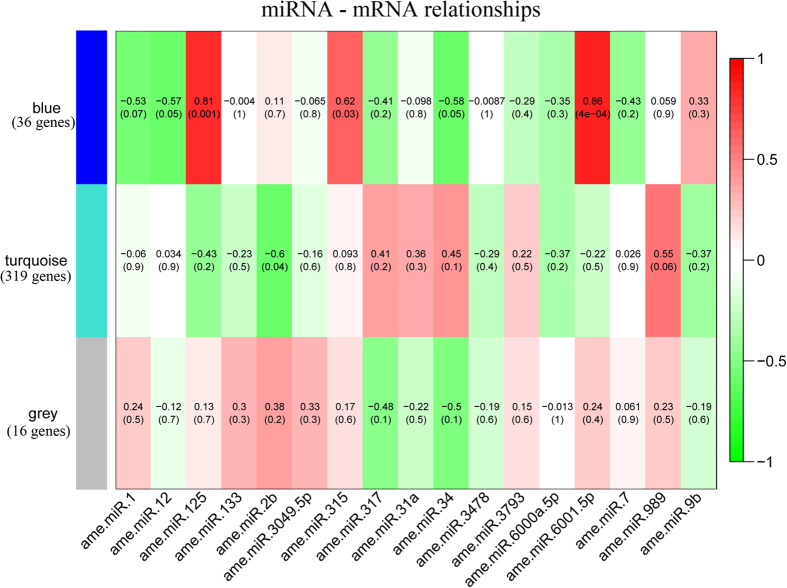
Associations between miRNAs and target genes at expression levels. By co-expression analysis, 371 target genes of 17 miRNAs were clustered into three groups. 36 target genes showed co-expression and were clustered into group 1 (blue). Another 319 target genes showed co-expression and were clustered into group 2 (turquoise). The remaining 16 target gene did not show co-expression were clustered into group 3 (grey). If the target prediction is true, the miRNAs should be correlated with target genes at the expression levels. We then quantified the correlation between the each of miRNAs with three groups of target genes. Each row corresponds to a co-expressed gene group and each column corresponds to a miRNA. Each cell contains the corresponding correlation and p-value. The table is color – coded by correlation according to the color legend on the right side.

By matching seed regions (2–8 bp) of mature miRNAs to 3′UTRs of honey bee genes, 413 predicted genes could be targeted by the 17 differentially expressed miRNAs. We expected a dynamic correlation between miRNA and its direct or indirect target mRNAs during the six day infection period. Therefore, we quantified the expression levels of honey bee transcripts in infected and uninfected honey bee workers from 1 to 6 dpi using RNA-seq data from NCBI bio-project PRJNA282511. Out of the 413 predicted gene targets, 371 were expressed according to three groups (Fig. 1). Group 1 (blue) and Group 2 (turquoise) consisted of 36 and 319 genes, respectively, and showed co-expression within each group. The remaining genes were clustered in a third (grey) group. Out of 17 miRNAs, 5 showed a significant correlation with group 1 (blue) and 2 miRNAs showed a significant correlation with group 2 (turquoise). No significant correlation was detected between miRNAs and group 3 (grey) (Fig. 1, Additional file 2).

Gene Ontology (GO) enrichment analysis was performed for the 371 expressed target genes to identify biologically significant terms. The top five ranked GO terms were iron binding (GO: 0043167), signaling (GO: 0023052), nucleus (GO: 0005634), DNA binding (GO: 0003677) and transmembrane transportation (GO: 0022857). Expressed target genes were also screened against the Kyoto Encyclopedia of Genes and Genomes (KEGG) database, from which nine biological pathways were identified. Out of these nine pathways, seven were metabolism related, including purine metabolism, pyrimidine metabolism, oxidative phosphorylation, amino sugar and nucleotide sugar metabolism (Table 2). 27 of the expressed target genes are predicted to encode enzymes involved in the nine KEGG pathways of which, 26 were clustered within group 2. Furthermore, 11 out of the 27 predicted enzymes were significantly correlated with ame-miR-2b expression (χ^2^ = 39.11, df = 1, p < 0.01), two of which are predicted targets of ame-miR-2b (LOC409904, LOC551826).

## Discussion

It is not uncommon for a single miRNA to regulate multiple genes or for multiple miRNAs to regulate the same gene^11^. Abnormal expression of miRNA could have subsequent effects on downstream gene expression. *N. ceranae* is known to change honey bee global gene expression^12^. From our data, early (1 dpi) and late infection (6 dpi) time points had a higher tendency for differential miRNA expression. This non-random distribution suggests that the honey bee miRNAs we identified might be related to particular phases of *N. ceranae* host invasion and reproduction. Handling stress during experimental inoculation may also explain early (1 dpi) differential gene expression.

From our data, the expression levels of 17 known miRNAs were differentially regulated during *N. ceranae* infection, which may target over 400 predicted genes for degradation. We found that transmembrane transporters were enriched in the target gene list. Transportation and metabolism GO categories were also previously found to be enriched for up-regulated genes in *N. ceranae* infected workers^13^. As *N. ceranae* reproduces within host midgut epithelial cells, regulating transmembrane transporters may control the availability of resources within *Nosema*-infected midgut cells, but how this affects the host or parasite remains to be determined. Increased transmembrane transportation would be more efficient for faster reproduction. We found purine metabolism, pyrimidine metabolism and oxidative phosphorylation pathways to be among the predicted functional roles of the miRNA target gene list. Within those pathways, 26 out of 27 predicted to encode enzymes showed co-expression and 25 had significantly correlated expression with the 17 differentially expressed miRNAs. Ame-miR-2b is particularly interesting because 11 out of 27 enzymes were significantly correlated with its expression level. It is known that *N. ceranae* infected honey bees consume significantly more sugar water^14^, indicative of increased metabolic need. The correlation between miRNAs and metabolism related genes may reflect a mechanism used by the parasite to alter host metabolism to fuel its faster replication. But the target genes are limited to computational prediction and require experimental confirmation. Our study is also limited by a general lack of information about 3′UTRs in microsporidian genes, including *N. ceranae*. Therefore empirical miRNA knock-down studies are required to verify the predicted target genes as well as the role of honey bee miRNAs on *N. ceranae* parasitism.

Additionally, honey bee and parasite strains need to be considered in order to conclude more broadly whether these differentially expressed genes are general responses toward parasite infection or are specific host responses to *N. ceranae*^15^,^16^,^17^,^18^. For example, *Nosema*-resistant bee strains have been developed and may be insightful models to study the molecular mechanisms specific to *Nosema* resistance^8^. The honey bee strain used in this study is not from a known *Nosema*-resistant lineage. Therefore, the honey bee responses we have identified are likely to be general responses toward *N. ceranae* infection and may differ from the mechanisms within *Nosema*-resistant strains.

This is the first study showing host miRNA responses toward microsproidian infections. Based on current data, it is unclear how *N. ceranae* infection leads to differentially expressed miRNAs. The key proteins that are important for the formation of small regulatory RNAs have also been reported from several protozoan parasites^19^,^20^,^21^. Fly gut defense against bacterial invasion depends in part on the RNAi component *Aubergine*^22^. In contrast, the fungus *Botrytis cinerea*, manipulates the host RNAi system to attack the host immune response, instead of suppressing its expression^2^. A previous study did not support that *N. ceranae* infection could suppress the expression level of honey bee RNAi genes^8^. The key genes that are involved in small regulatory RNA synthesis have been identified from the *N. ceranae* genome^23^. We hypothesize that the parasite might generate and secrete small regulatory RNAs into the cytoplasm to interfere with host miRNAs. Alternatively, small regulatory RNAs from the parasite might directly target honey bee genes, an excellent topic for future work.

## Materials and Methods

### Ethics statement

The apiaries for bee sample collection were maintained by the USDA-ARS Bee Research Laboratory, Beltsville, Maryland, USA. No specific permits were required for the described studies. Studies involved the European honey bee (*Apis mellifera*), which is neither an endangered nor protected species.

### Infection and Sampling

*N. ceranae* spores were isolated from the midguts of worker honey bees in heavily infected colonies and purified using a Percoll gradient procedure^23^. Spores were counted using a Fuchs-Rosenthal haemocytometer and *N. ceranae* species status was verified by a standard PCR protocol^16^. 150 newly emerged workers were individually fed with 2 μl 50% sucrose solution containing 10^5^ *N. ceranae* spores. An additional 150 newly emerged workers were individually fed with 2 μl sucrose solution without spores, as a control. Three sets of 50 workers each for control and infected bees were housed in a sterile plastic cup at 34 ± 1 °C, 60% relative humidity. Five workers were sampled from each cup at 24 hour intervals for six days post-infection. The midguts of all sampled workers were used to extract total RNA with TRIzol. Total RNA of 15 infected and 15 control workers respectively were pooled daily and small RNA were separated with small RNA sample Prep Kit for ILLUMINA sequencing. The remaining of the total RNA was used for mRNA sequencing and the original mRNA sequencing data were downloaded from NCBI bio-project PRJNA282511. One small RNA library was sequenced for each post infection day. Overall, six libraries were sequenced for infected workers and an additional six libraries were sequenced for the control workers. The purpose of this study is searching the miRNA expression variance due to the infection. All the honey bee workers were from the same colony. The main reason to use one colony is to assure that the sampled bees were not a mix of recalcitrant bees with completely unsuccessful infections and bees for which infection was possible, as this would have confounded the control vs. infection comparisons.

### MicroRNA data analysis

Over 14 million reads (19–35bp per read) were generated from each small RNA library, respectively, after quality control. The small RNA reads were aligned to the *A. mellifera* genome (Amel 4.5) to extract the counts of mature miRNAs via the miRdeep2 package^24^. The original counts were normalized with weighted trimmed mean of M-values (TMM) to calculate relative expression levels of miRNA using edgeR^25^. As there is no library replicate, the common dispersion 0.02 was used to calculate corrected P value of each miRNAs. miRNAs with fewer than 100 normalized reads across all 12 libraries were discarded. We define significantly differentially expressed miRNAs as those with both a fold-change of expression >2 and p value < 0.05. Annotated 3’UTR of honey bee protein coding genes were used for miRNA target prediction using Miranda package with an energy threshold of 20^26^. We then used the protein sequences of predicted target genes to retrieve GO terms using Blast2GO software^27^. Biological pathways were retrieved from KEGG database by screening the predicted target genes. The Pearson association between miRNAs and their target genes at the expression level were analyzed by WGCNA package^28^. Original small RNA sequencing data have been uploaded to NCBI bio-project PRJNA282511.

### MicroRNA data verification with RT-qPCR

The same total RNA that was used to construct the sequencing library was used to synthesis cDNA with miRCURY LNA^TM^ Universal RT kit (EXIQON). The reverse transcription reaction setup is revised from the factorial instruction by increasing the input total RNA to 1000 ng. The primers for miRNA were designed based on the mature sequence and EciLENT SYBR Green master mix (EXIQON) was used for RT-qPCR reaction according to factory instruction. We randomly selected 5 miRNAs (ame-miR-12, ame-miR-315, ame-miR-317, ame-miR-31a, ame-miR34) for RT-qPCR assays. The amplification efficiency for each miRNA primer was calculated with qpcR package^29^. Ame-miR-12 is used as reference to calculate the relative expression level of the other four miRNAs. The correlation of their expression levels between sequencing data and RT-qPCR data was calculated by SPSS package for data validation.

## Additional Information

**How to cite this article**: Huang, Q. *et al.* Honey bee microRNAs respond to infection by the microsporidian parasite *Nosema ceranae*. *Sci. Rep.* **5**, 17494; doi: 10.1038/srep17494 (2015).

## Supplementary Material

Supplementary Information

Additional file 1

Additional file 2

## Figures and Tables

**Table 1:** Proportion of normalized reads from 17 significantly differentially expressed miRNAs in infected bees at each time point (y = count^infected workers^/(count^infected workers^ + count^control workers^)).

MiRNAs	1 dpi	2 dpi	3 dpi	4 dpi	5 dpi	6 dpi
ame-miR-1	0.553	0.518	0.505	0.414	0.554	0.355*
ame-miR-12	0.44	0.511	0.555	0.494	0.671**	0.359*
ame-miR-125	0.536	0.453	0.604	0.533	0.511	0.328**
ame-miR-133	0.602	0.535	0.474	0.476	0.453	0.345*
ame-miR-2b	0.545	0.59	0.505	0.519	0.504	0.192**
ame-miR-3049-5p	0.513	0.458	0.447	0.647	0.508	0.288**
ame-miR-315	0.329*	0.493	0.399	0.525	0.343*	0.610
ame-miR-317	0.643	0.622	0.54	0.535	0.728**	0.514
ame-miR-31a	0.486	0.582	0.539	0.548	0.3**	0.675**
ame-miR-34	0.663*	0.533	0.552	0.509	0.542	0.493
ame-miR-3478	NA	0**	NA	1**	NA	NA
ame-miR-3793	NA	NA	NA	0**	0**	NA
ame-miR-6000a-5p	0.657	0.471	0.509	0.569	0.563	0.187**
ame-miR-6001-5p	0.242**	0.336	0.547	0.404	0.392	0
ame-miR-7	0.725**	0.606	0.495	0.482	0.649	0.279**
ame-miR-989	0.463	0.458	0**	NA	0.427	0.516
ame-miR-9b	1**	0.514	NA	1	NA	NA

Values < 0.5 indicate down-regulated mean expression. NA: miRNA was not detected in control or infected workers at the sampling point. Significant differential expression at < 0.05 (*) and < 0.01 (**).

**Table 2:** Number of genes in the top five ranked GO terms and top 9 KEGG pathways.

		Number of genes
GO Terms	Iron binding (GO: 0043167)	92
	Signaling (GO: 0023052)	35
	Nucleus (GO: 0005634)	31
	DNA binding (GO: 0003677)	21
	Transmembrane transporter activity (GO: 0022857)	21
KEGG Pathways	Purine metabolism	6
	Thiamine metabolism	1
	Aminobenzoate degradation	2
	Biosysthesis of antibiotics	4
	Oxidative phosphorylation	3
	Glycan degradation	2
	Riboflavin metabolism	1
	Amino sugar and nucleotide sugar metabolism	2
	Pyrimidine metabolism	2
