# Supplementary material for: Honey bee microRNAs respond to infection by the microsporidian parasite Nosema ceranae
Source: Sci Rep. 2015 Dec 1;5:17494. doi: 10.1038/srep17494 (PMC4664923; doi:10.1038/srep17494)
Supplement: Supplementary Information [file srep17494-s1.doc]

Honey bee microRNAs respond to infection by the microsporidian parasite *Nosema ceranae*

Qiang Huang^1,2*^, Yanping Chen^2^, Rui Wu Wang^1*^, Ryan S. Schwarz^2^, Jay D. Evans^2*^

^1^State Key Laboratory of Genetic Resources and Evolution, Kunming Institute of Zoology, Chinese Academy of Science, Kunming, 650223, China.

^2^USDA-ARS Bee Research Laboratory, BARC-East Building 306, Beltsville, Maryland, 20705, USA.

Table S1 Ct values and amplification efficiency for RT-qPCR assay

| Efficiency | 2 | 2 | 2 | 2 | 2 | 1.8855 | 1.8282 |
| --- | --- | --- | --- | --- | --- | --- | --- |
|  | ame-miR-12 | ame-miR-315 | ame-miR-317 | ame-miR-31a | ame-miR-34 | NCER_100079 | NCER_101240 |
| Day1_control | 18.76 | 26.23 | 24.83 | 22.12 | 24.37 | NA | NA |
| Day2_control | 18.22 | 26.10 | 23.36 | 21.49 | 22.97 | NA | NA |
| Day3_control | 17.49 | 25.07 | 22.27 | 21.3 | 21.83 | NA | NA |
| Day4_control | 17.77 | 25.40 | 22.24 | 21.66 | 22.01 | NA | NA |
| Day5_control | 17.91 | 24.90 | 22.10 | 20.38 | 21.54 | NA | NA |
| Day6_control | 18.30 | 27.12 | 23.59 | 21.91 | 23.76 | NA | NA |
| Day1_infection | 19.40 | 27.31 | 24 | 21.96 | 23.55 | 40.82 | 34.01 |
| Day2_infection | 18.26 | 26.26 | 23.06 | 21.16 | 22.95 | 42.56 | 32.81 |
| Day3_infection | 17.33 | 24.68 | 21.48 | 20.34 | 21.04 | 32.06 | 28.47 |
| Day4_infection | 17.26 | 24.84 | 21.73 | 20.53 | 21.36 | 35.22 | 25.10 |
| Day5_infection | 17.49 | 25.05 | 21.68 | 20.88 | 20.92 | 33.39 | 21.68 |
| Day6_infection | 17.52 | 25.68 | 22.62 | 21.01 | 22.15 | 33.6 | 20.24 |


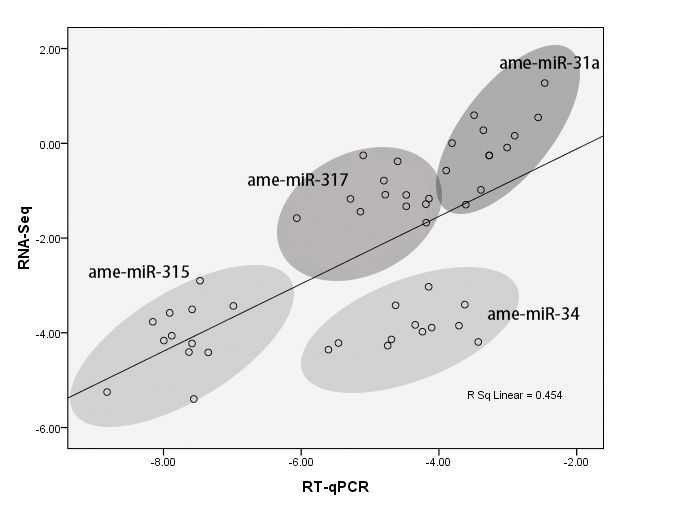


Figure S1 correlation between RNA-seq data and RT-qPCT data. 5 miRNAs were tested with RT-qPCR and the relative expression value was normalized with ame-miR-12 for each library. The expression data RNA-seq is significantly correlated with RT-qPCR (p < 0.05).
